# Supplementary material for: High rates of apoptosis visualized in the symbiont-bearing gills of deep-sea Bathymodiolus mussels
Source: PLoS One. 2019 Feb 4;14(2):e0211499. doi: 10.1371/journal.pone.0211499 (PMC6361440; doi:10.1371/journal.pone.0211499)
Supplement: S1 Table — (DOCX) [file pone.0211499.s001.docx]

**Supplementary Table 1**

| Species | Site | Recovery | Cruise | ID | Length in mm | Width in mm | Thickness in mm | % Apoptosis median | % Apoptosis SD |
| --- | --- | --- | --- | --- | --- | --- | --- | --- | --- |
| *Bathymodiolus azoricus* | Menez Gwen | pressurised | BioBaz 2013 | 17 | 34 | 20 | 14 | 31.0% | 15.4% |
| *Bathymodiolus azoricus* | Menez Gwen | pressurised | BioBaz 2013 | 18 | 37 | 21 | 15 | 22.6% | 12.2% |
| *Bathymodiolus azoricus* | Menez Gwen | pressurised | BioBaz 2013 | 20 | 32 | 18.3 | 11.2 | 39.8% | 13.0% |
| *Bathymodiolus azoricus* | Menez Gwen | pressurised | BioBaz 2013 | 22 | 26.3 | 14.7 | 9.3 | 22.5% | 9.6% |
| *Bathymodiolus azoricus* | Menez Gwen | pressurised | BioBaz 2013 | 24 | 30.3 | 17.6 | 10.2 | 52.8% | 19.5% |
| *Bathymodiolus azoricus* | Menez Gwen | unpressurised | BioBaz 2013 | 27 | 42.5 | 23.2 | 16 | 80.4% | 31.4% |
| *Bathymodiolus azoricus* | Menez Gwen | unpressurised | BioBaz 2013 | 29 | 41 | 20.3 | 16.2 | 11.6% | 13.7% |
| *Bathymodiolus azoricus* | Menez Gwen | unpressurised | BioBaz 2013 | 30 | 41 | 21 | 15 | 20.8% | 13.4% |
| *Bathymodiolus azoricus* | Menez Gwen | unpressurised | BioBaz 2013 | 31 | 37.3 | 20 | 13.5 | 4.2% | 26.5% |
| *Bathymodiolus azoricus* | Menez Gwen | unpressurised | BioBaz 2013 | 33 | 32 | 16.8 | 13 | 14.4% | 11.7% |
| *Bathymodiolus azoricus* | Rainbow | pressurised | BioBaz 2013 | 130 | 88.5 | 37.5 | 32 | 28.3% | 13.7% |
| *Bathymodiolus azoricus* | Rainbow | pressurised | BioBaz 2013 | 131 | 83 | 39.5 | 33.5 | 55.3% | 25.0% |
| *Bathymodiolus azoricus* | Rainbow | pressurised | BioBaz 2013 | 132 | 79 | 35 | 30.5 | 37.3% | 19.7% |
| *Bathymodiolus azoricus* | Rainbow | pressurised | BioBaz 2013 | 133 | 82.5 | 36.5 | 31 | 13.1% | 14% |
| *Bathymodiolus azoricus* | Rainbow | pressurised | BioBaz 2013 | 134 | 84 | 37 | 31.5 | 21.9% | 14.5% |
| *Bathymodiolus azoricus* | Rainbow | pressurised | BioBaz 2013 | 135 | 67 | 33.5 | 25 | 26.7% | 15.7% |
| *Bathymodiolus azoricus* | Rainbow | pressurised | BioBaz 2013 | 136 | 86 | 41 | 32 | 50.3% | 25.4% |
| *Bathymodiolus azoricus* | Rainbow | pressurised | BioBaz 2013 | 137 | 70.5 | 33.5 | 29 | 6.3% | 6.9% |
| *Bathymodiolus azoricus* | Rainbow | unpressurised | BioBaz 2013 | 141 | 97 | 41 | 37 | 39.4% | 11.8% |
| *Bathymodiolus azoricus* | Rainbow | unpressurised | BioBaz 2013 | 142 | 84 | 41.5 | 38 | 36.5% | 15.0% |
| *Bathymodiolus azoricus* | Rainbow | unpressurised | BioBaz 2013 | 143 | 88 | 41 | 35 | 12.4% | 7.9% |
| *Bathymodiolus puteoserpentis* | Snake pit | unpressurised | BICOSE 2014 | 102 | 125 | 60 | 44 | 15.9% | 9.8% |
| *Bathymodiolus puteoserpentis* | Snake pit | unpressurised | BICOSE 2014 | 105 | 89 | 42 | 33 | 26.0% | 15.3% |
| *Bathymodiolus puteoserpentis* | Snake pit | pressurised | BICOSE 2014 | 203 | 52 | 30 | 22 | 40.9% | 25.5% |
| *Bathymodiolus puteoserpentis* | Snake pit | pressurised | BICOSE 2014 | 501 | 117 | 56 | 47 | 40.9% | 25.5% |
| *Bathymodiolus puteoserpentis* | Snake pit | pressurised | BICOSE 2014 | 502 | 91 | 47 | 37 | 28.7% | 16.1% |
| *Bathymodiolus puteoserpentis* | Snake pit | pressurised | BICOSE 2014 | 504 | 41 | 24 | 17 | 6.03% | Ø |
| *Bathymodiolus puteoserpentis* | Snake pit | pressurised | BICOSE 2014 | 511 | 67 | 39 | 31 | 18.4% | Ø |
| *Bathymodiolus puteoserpentis* | Snake pit | pressurised | BICOSE 2014 | 514 | Ø | Ø | Ø | 31.2% | 7.7% |
| *Bathymodiolus puteoserpentis* | Snake pit | unpressurised | BICOSE 2014 | 515 | 83 | 44 | 37 | 11.4% | Ø |
| *Bathymodiolus puteoserpentis* | Snake pit | unpressurised | BICOSE 2014 | 522 | Ø | Ø | Ø | 16.4% | 14.5% |
| *Bathymodiolus boomerang* | M2 | unpressurised | WACS 2011 | 6 | 87 | 31 | 23 | 11.3% | 12.7% |
| *Bathymodiolus boomerang* | M2 | unpressurised | WACS 2011 | 7 | 113 | 31 | 23 | 26.3% | 13.0% |
| *Bathymodiolus boomerang* | M2 | unpressurised | WACS 2011 | 8 | 154 | 48 | 37 | 31.1% | Ø |
| *Bathymodiolus boomerang* | M2 | unpressurised | WACS 2011 | 9 | 162 | 46 | 43 | 7.5% | 19.9% |
| *Bathymodiolus boomerang* | M2 | unpressurised | WACS 2011 | 10 | 31 | 16 | 11 | 23.9% | 18.8% |
| *Bathymodiolus boomerang* | M1 | unpressurised | WACS 2011 | 27 | 64 | 24 | 18 | 12.5% | 7.9% |
| *Bathymodiolus boomerang* | M1 | unpressurised | WACS 2011 | 28 | 74 | 31 | 20 | 8.2% | 3.2% |
| *Bathymodiolus boomerang* | M1 | unpressurised | WACS 2011 | 30 | 117 | 35 | 27 | 24.8% | 24.0% |
| *Bathymodiolus boomerang* | M2 | unpressurised | WACS 2011 | 9H | 127 | 43 | 36 | 17.0% | 12.8% |
| *Mytilus edulis* | Roscoff |  |  | 6 | 17 | 23 | 14 | 1.7% | 0.8% |
| *Mytilus edulis* | Roscoff |  |  | 4 | 23 | 43 | 18 | 1.6% | 1.2% |
| *Mytilus edulis* | Roscoff |  |  | 5 | 22 | 37 | 16 | 1.0% | Ø |
| *Mytilus edulis* | Roscoff |  |  | 7 | 14 | 23 | 10 | 1.1% | Ø |
| *Mytilus edulis* | Fishmonger |  |  | 11 | 53 | Ø | Ø | 2.0% | 2.3% |
| *Mytilus edulis* | Fishmonger |  |  | 12 | 48 | Ø | Ø | 0.7% | 0.3% |
| *Mytilus edulis* | Fishmonger |  |  | 13 | 51 | Ø | Ø | 1.0% | Ø |
| *Mytilus edulis* | Fishmonger |  |  | 14 | 56 | Ø | Ø | 2.2% | 0.8% |
| *Mytilus edulis* | Fishmonger |  |  | 15 | 49 | Ø | Ø | 2.0% | 2.2% |
