# Supplementary figures and images for: High rates of apoptosis visualized in the symbiont-bearing gills of deep-sea Bathymodiolus mussels
Source: PLoS One. 2019 Feb 4;14(2):e0211499. doi: 10.1371/journal.pone.0211499 (PMC6361440; doi:10.1371/journal.pone.0211499)

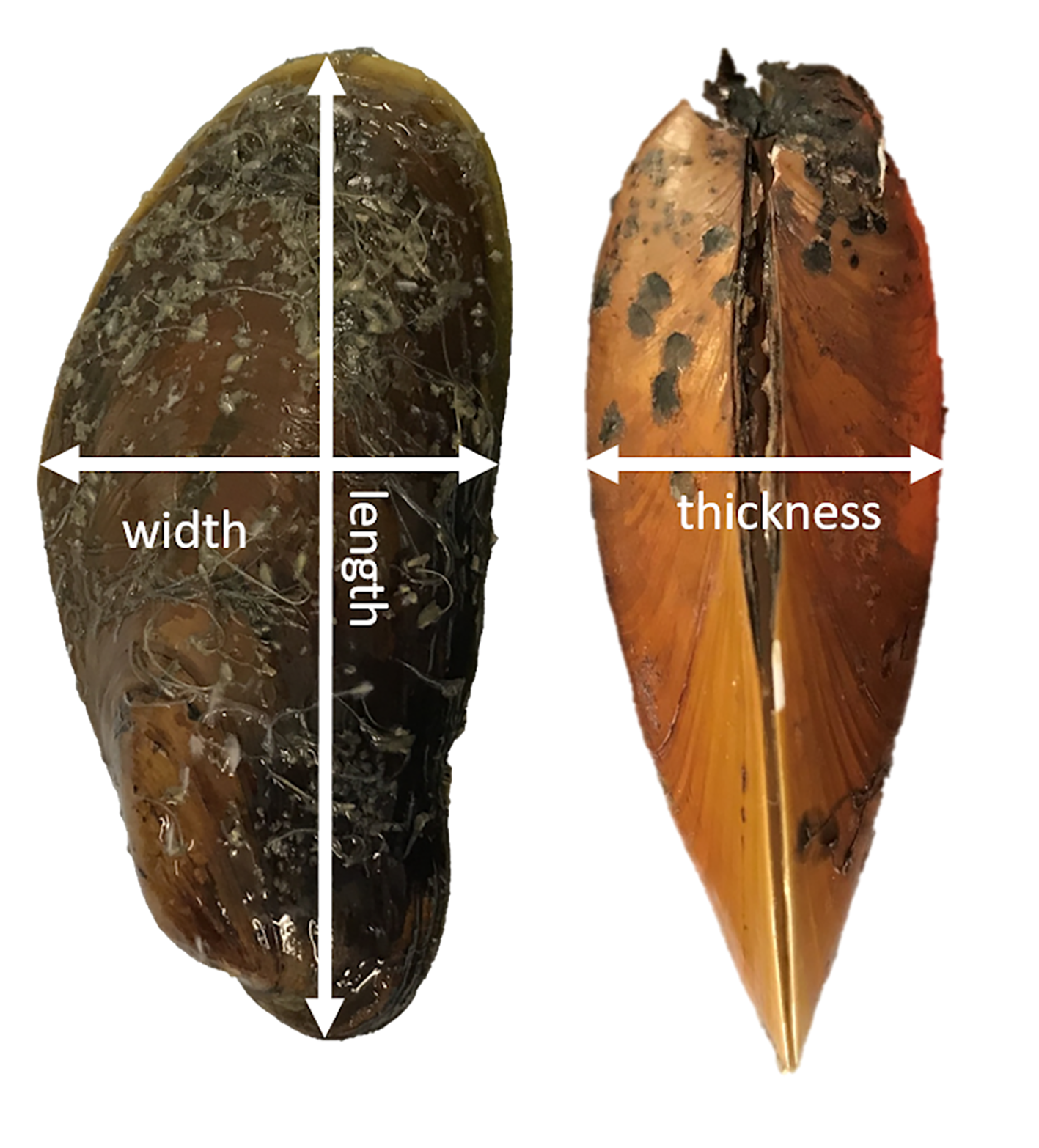

Supplement: S1 Fig — Using a caliper, the length, width and thickness of each individual was taken as shown on the figure, and recorded in S1 Table. (TIF) [file pone.0211499.s002.tif]

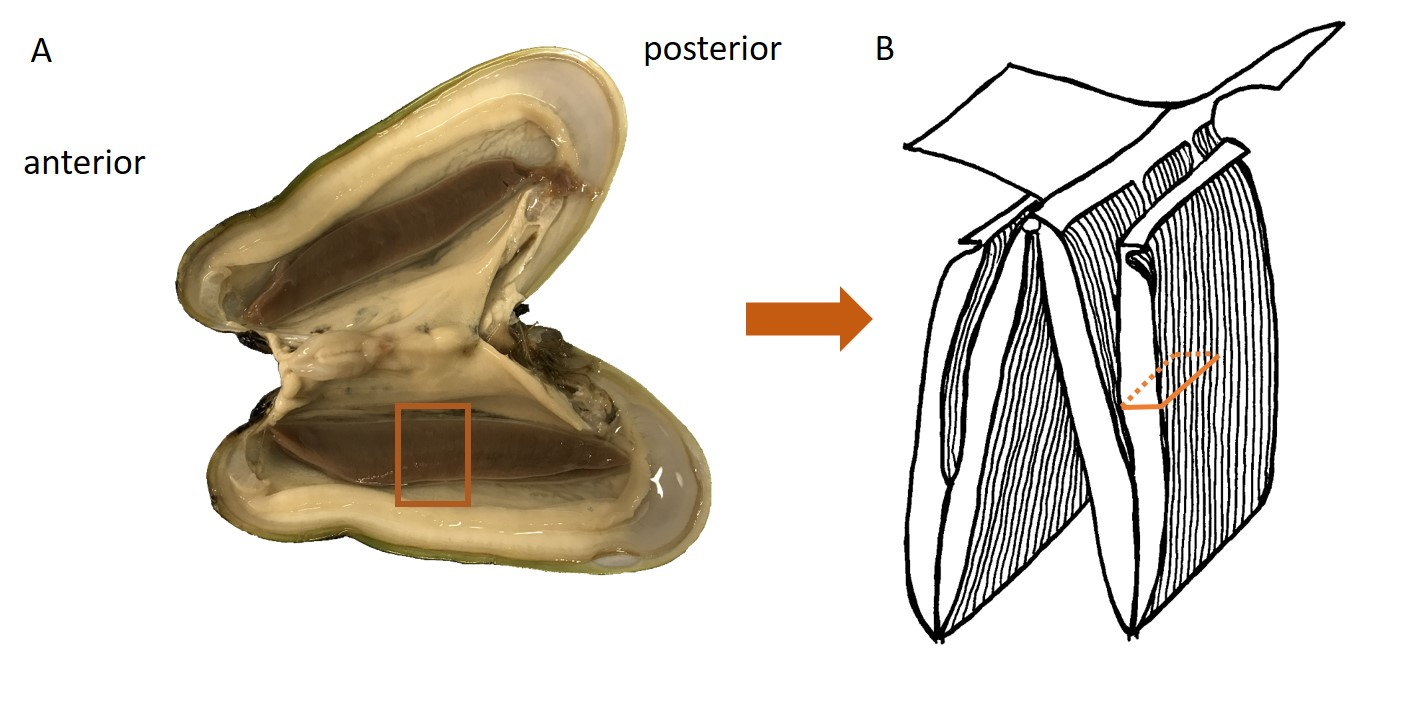

Supplement: S2 Fig — A: Bathymodiolus azoricus with open mantle cavity showing its two gills. The frame corresponds to the anterior gill part used in this study. B: Gills from one side (adapted from [70] showing the unfused W-shaped pairs of demibranchs and the ventral-transversal plane of sectioning across the gill lamellae (orange framed box). (TIFF) [file pone.0211499.s003.tiff]

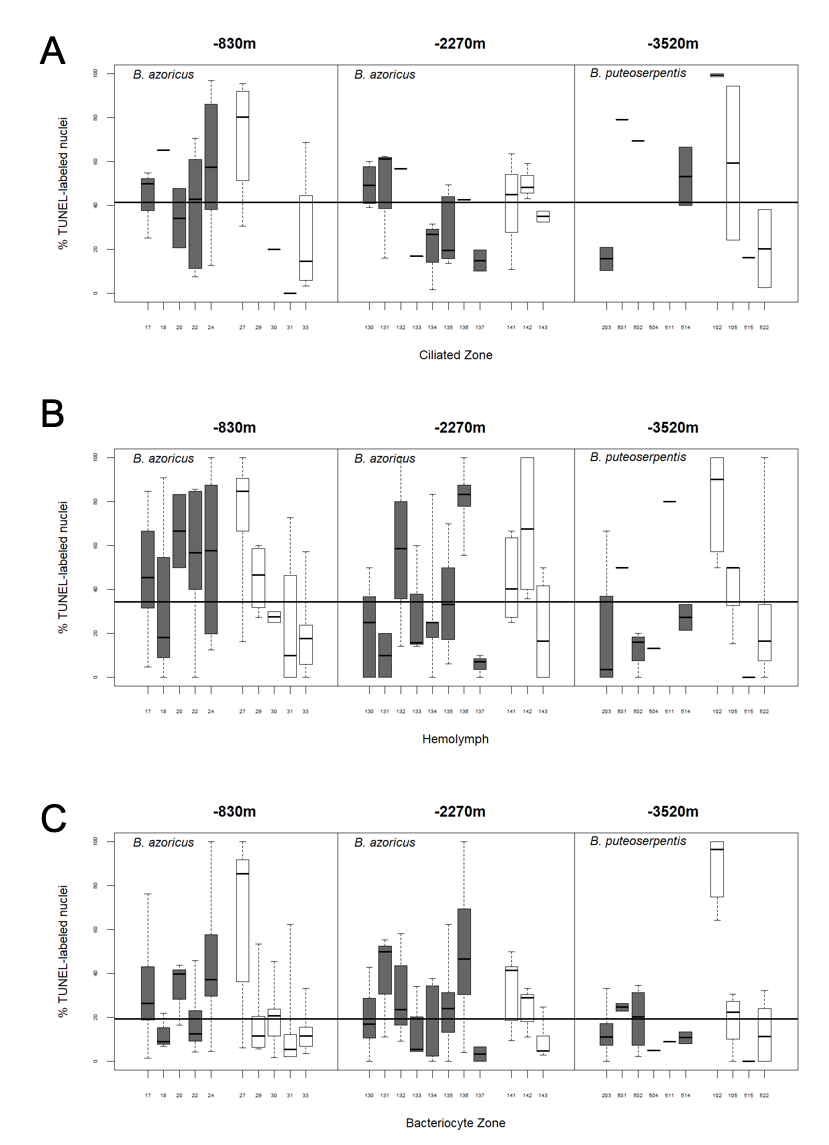

Supplement: S3 Fig — Percentage of apoptotic nuclei in the ciliated (A), hemolymph (B) and bacteriocyte (C) zones in individual specimens of B. azoricus and B. puteoserpentis from the three sites. White and grey boxplots indicate specimens from non-isobaric and isobaric recoveries, respectively. Boxplot whiskers indicate minimal and maximal values of percentage of TUNEL-labelled nuclei on a single image, the line inside the box is the median, the line inside the box is the median, and the upper and lower frames of the boxes represent the first and third quartile respectively. The bold horizontal line through all boxplots represents the median of all pictures. The numbers below the Y-axis of the figures correspond to the specimen ID. (TIFF) [file pone.0211499.s004.tiff]

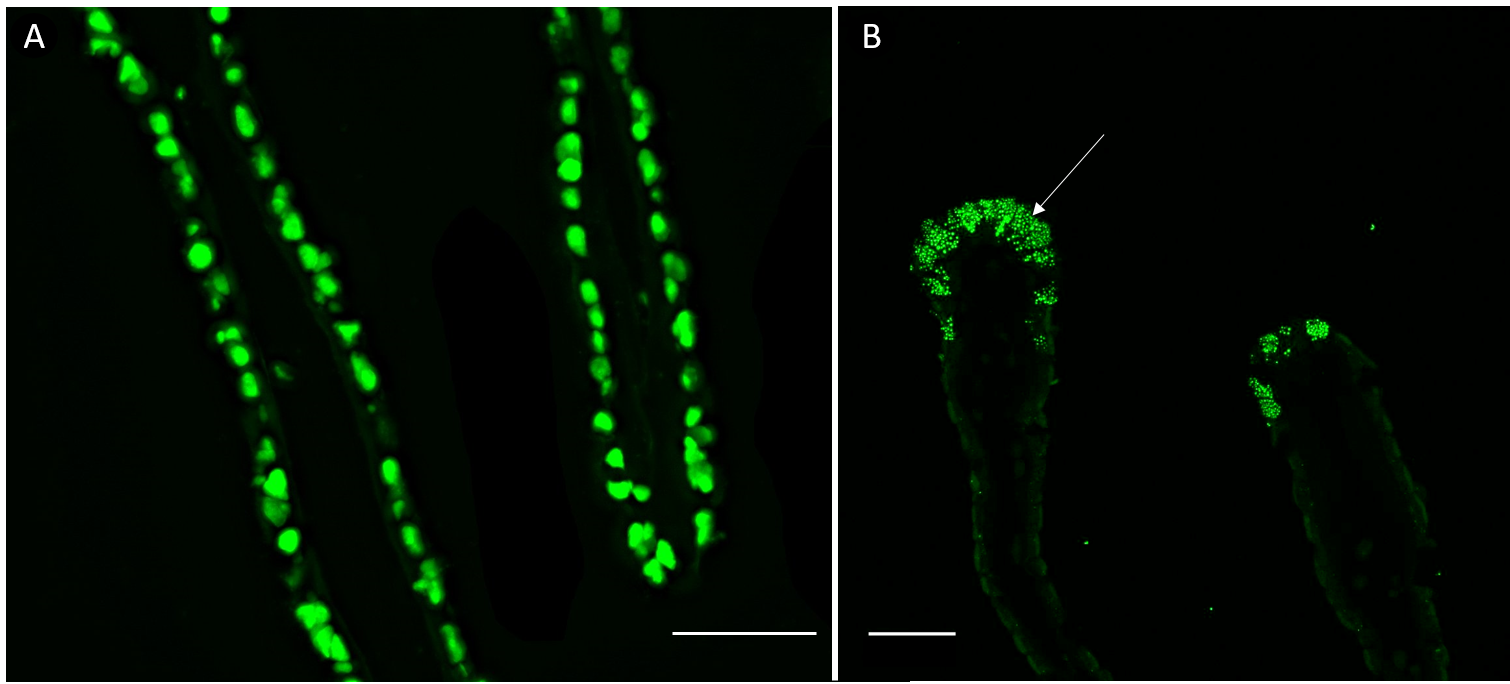

Supplement: S4 Fig — A. Positive control with all nuclei labelled (in green). B. Negative control with non-specific autofluorescent putative mucus-like granules (arrow). Scale bars: 50μm. (TIF) [file pone.0211499.s005.tif]

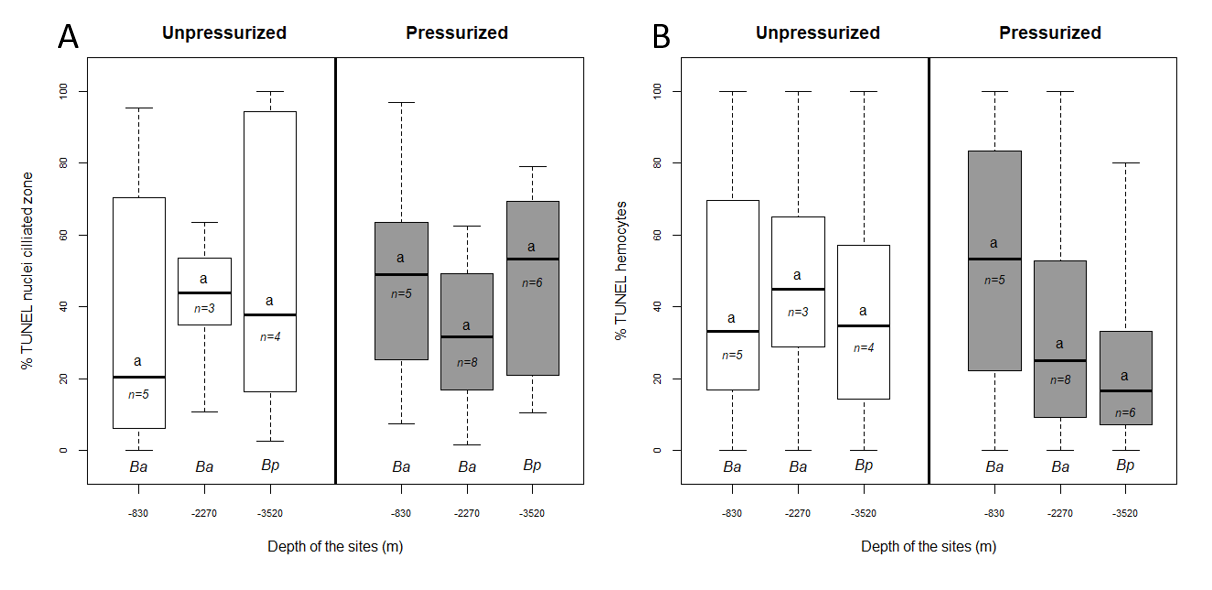

Supplement: S5 Fig — Percentage of apoptotic nuclei in the ciliated (A) and hemolymph zones (B) of B. azoricus and B. puteoserpentis from the three sites. Grey and white boxplots indicate specimens from isobaric and non-isobaric recoveries, respectively. No significant differences were seen among groups (Pairwise Wilcoxon with Bonferroni’s standard correction). Boxplot whiskers indicate minimal and maximal values on a single image, line inside the box is the median, and the upper and lower frames of the boxes represent the first and third quartiles respectively. (TIFF) [file pone.0211499.s006.tiff]
